# Protein Phosphatase-1α Interacts with and Dephosphorylates Polycystin-1

**DOI:** 10.1371/journal.pone.0036798

**Published:** 2012-06-04

**Authors:** Stephen C. Parnell, Sanjeev Puri, Darren P. Wallace, James P. Calvet

**Affiliations:** 1 Department of Biochemistry and Molecular Biology, University of Kansas Medical Center, Kansas City, Kansas, United States of America; 2 Kidney Institute, University of Kansas Medical Center, Kansas City, Kansas, United States of America; 3 Biotechnology Department, University Institute of Engineering and Technology, Panjab University, Chandigarh, India; 4 Department of Medicine and the Kidney Institute, University of Kansas Medical Center, Kansas City, Kansas, United States of America; University of Cambridge, United Kingdom

## Abstract

Polycystin signaling is likely to be regulated by phosphorylation. While a number of potential protein kinases and their target phosphorylation sites on polycystin-1 have been identified, the corresponding phosphatases have not been extensively studied. We have now determined that polycystin-1 is a regulatory subunit for protein phosphatase-1α (PP1α). Sequence analysis has revealed the presence of a highly conserved PP1-interaction motif in the cytosolic, C-terminal tail of polycystin-1; and we have shown that transfected PP1α specifically co-immunoprecipitates with a polycystin-1 C-tail construct. To determine whether PP1α dephosphorylates polycystin-1, a PKA-phosphorylated GST-polycystin-1 fusion protein was shown to be dephosphorylated by PP1α but not by PP2B (calcineurin). Mutations within the PP1-binding motif of polycystin-1, including an autosomal dominant polycystic kidney disease (ADPKD)-associated mutation, significantly reduced PP1α-mediated dephosphorylation of polycystin-1. The results suggest that polycystin-1 forms a holoenzyme complex with PP1α via a conserved PP1-binding motif within the polycystin-1 C-tail, and that PKA-phosphorylated polycystin-1 serves as a substrate for the holoenzyme.

## Introduction

Phosphorylation is a common post-translational modification of proteins that affects their structure and ability to interact with other proteins, and thus ultimately their function. As such, understanding the functions of protein kinases and phosphatases is of critical importance to cellular biology. Protein phosphatase-1 (PP1) is a widely expressed serine/threonine phosphatase that regulates numerous cellular functions including ion channel activity, cytoskeletal organization, cell cycle progression, and gene transcription [1], [2]. The broad substrate specificity of PP1 is dictated by its interactions with a wide range of regulatory proteins [3]. These “holoenzyme” complexes can dephosphorylate single or multiple substrates and are themselves subject to being regulated by events such as post-translational modification or binding of additional accessory proteins [1], [2], [4]. The mammalian genome contains three genes encoding four isoforms of PP1 (α, β, γ_1_, and γ_2_) that are approximately 90% identical. However, differences within their N- and C-termini affect binding to regulatory proteins and are important for enzyme specificity [2].

PP1-binding regulatory proteins are structurally and functionally diverse. Most regulatory proteins contain an “RVxF” motif (where R can be arginine or lysine and x  =  any amino acid) that binds within a hydrophobic groove located far away from the active site of the phosphatase [5]. The motif “FxxBxB” (where B  =  basic) has also been identified as a PP1-binding domain [6], [7]; and recently additional regulatory protein-binding regions within PP1 have been identified [8]. While all PP1-regulatory proteins localize the phosphatase to specific regions within the cell, some also directly affect the catalytic activity of the enzyme.

Autosomal dominant polycystic kidney disease (ADPKD) is a common genetic disorder affecting 1∶400–1,000 individuals and leading to ∼10% of all end-stage renal disease [9], [10]. The hallmark of ADPKD is the presence of many fluid-filled renal cysts. Proliferation and expansion of these cysts leads to the progressive loss of renal function, with roughly half of the disease population undergoing renal failure by the sixth decade of life [11]. Extrarenal manifestations include hypertension, cardiac hypertrophy, hepatic and pancreatic cysts, and cerebral aneurysms [12], [13].

Mutations in either *PKD1* or *PKD2* cause ADPKD, suggesting that the protein products of these genes, polycystin-1 (PC1) and polycystin-2 (PC2) are functionally linked. Indeed, PC2 interacts with PC1 via a probable coiled-coil domain, and disruption of this interaction is thought to be sufficient to cause PKD [14]. PC2 forms a non-selective, calcium permeable cation channel, and is thought to regulate intracellular calcium levels in response to mechanical stimulation, cell adhesion, or cell surface receptor activation [15]. PC1 is a membrane-associated protein with a large N-terminal extracellular domain of about 3,000 amino acids, 11 transmembrane domains, and a C-terminal cytosolic domain of about 200 amino acids [16]. PC1 has been implicated in the regulation of a number of cellular pathways, including heterotrimeric G protein, nuclear factor of activated T-cells (NFAT), AP-1, β-catenin, mTOR, and intracellular calcium signaling [17], [18], [19]. However, the mechanism(s) by which PC1 is able to control such diverse signaling events remains unclear.

In the current study, we demonstrate that PC1 contains a conserved RVxF motif, that PP1α interacts with and dephosphorylates PC1, and that PC1 mutations within the RVxF motif, including an ADPKD-associated mutation, block PP1α-mediated dephosphorylation. Our results suggest that PC1 and PP1α interact to form a holoenzyme complex. We speculate that this complex may regulate certain aspects of PC1-mediated signaling.

## Materials and Methods

### Plasmids

**Figure 1:**
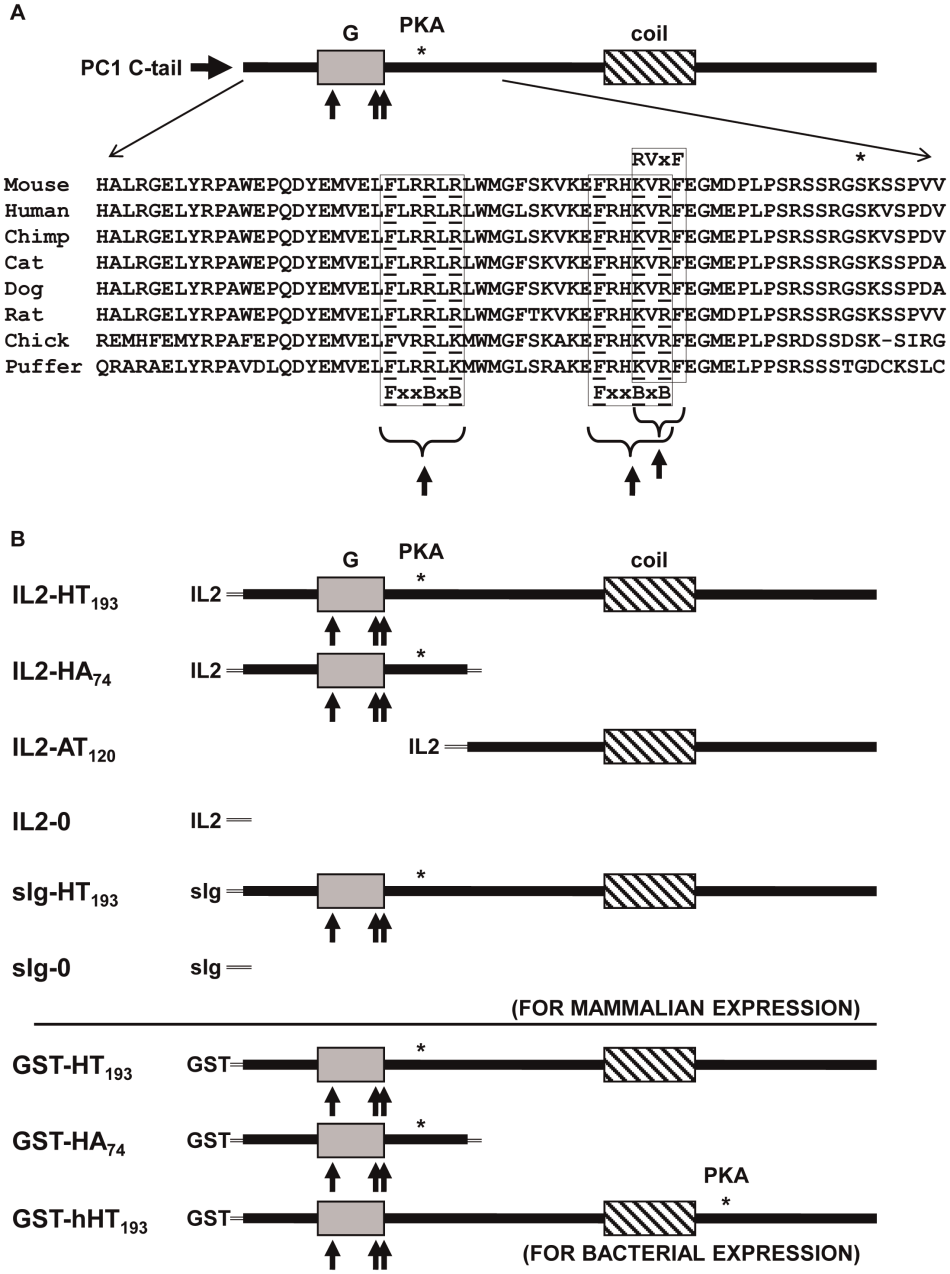
Polycystin-1 (PC1) sequence alignment and fusion proteins. (A) Putative protein phosphatase 1- (PP1) binding motifs (RVxF and FxxBxB; where R can be arginine or lysine, B  =  basic and x  =  any amino acid) were identified in the C-terminal, cytosolic tail of mouse PC1 by visual inspection of the primary amino acid sequence. Sequence alignment of a 65 amino acid region spanning the residues of interest reveals conservation of the putative PP1-binding motifs (boxed residues, up arrows) amongst PC1 homologs of diverse species from puffer fish to mouse. The location of the aligned sequence and putative binding motifs in the C-tail of mouse PC1 are shown schematically at the top. Additional features of the C-tail are indicated, including the G protein binding and activation domain (gray box, labeled “G”), the coiled-coil domain (hatched box, labeled “coil”), and PKA phosphorylation sites identified in PC1 (*, labeled “PKA”). (B) DNA constructs encoding IL2-, sIg-, and GST-PC1 fusion proteins were generated for expression in mammalian (IL2 and sIg) and bacterial cells (GST). All PC1 sequences are from mouse unless indicated. The IL2 constructs encode the N-terminal 269 amino acids comprising the complete extracellular and transmembrane domain of the α chain of the human IL2 receptor, followed by polylinker amino acids isoleucine-proline fused to PC1 C-tail sequences. sIg constructs have been described previously [21]. The GST constructs encode glutathione-S-transferase followed by PC1 C-tail sequences. The HT_193_ portion contains the complete C-terminal 193 amino acids of mouse PC1 (amino acids H4101 to T4293). hHT_193_ (bacterial expression only) contains the homologous C-terminal 193 amino acids of human (h) PC1 (amino acids H4110 to T4302). HA_74_ contains the putative PP1-binding motif, G protein activation domain, and PKA phosphorylation site; but lacks the coiled-coil domain. AT_120_ contains the coiled-coil domain, but lacks the features noted for the HA_74_ protein. The negative control, IL2-0 construct encodes the 269 amino acids of the IL2 receptor followed by vector-encoded amino acids ILQISITLAAARACI.

pBlueScript was from Stratagene. pKHA3-PP1α was generously provided by David L. Brautigan and was described previously [20]. pcDNA1.1/AMP IL2-0 contains an EcoRI fragment encoding the N-terminal 269 amino acids of the α-chain of the IL2 receptor (derived from pXS Tac Glut4*; generously provided by Nat Wolins; *a BglII site has been introduced in this construct, leading to the mutation of amino acids 238–239 from EY to DL) subcloned into pcDNA1.1/AMP (Invitrogen). pcDNA1.1/AMP IL2-fusions were produced by subcloning EcoRI-NotI *Pkd1* fragments encoding HT_193_, HA_74_, and AT_120_ (see Figure 1) in-frame with the IL2 receptor encoding portion of pcDNA1.1/AMP IL2-0. The complete coding sequence and 3′ UTR of IL2-HT_193_ used in these studies is shown in Figure S1A. The HA_74_ and AT_120_ fragments were produced by PCR amplification with their respective forward and reverse primer pairs (Figure S1B). pGEX4T1-HT_193_ and -HA_74_ were described previously [17]. To produce the human (h) GST-polycystin-1 fusion protein construct, pGEX4T1-hHT_193_, the region encoding the C-terminal 193 amino acids and stop codon of human *PKD1* was PCR-amplified using primers hHT-forward and AT-reverse (Figure S1B) and cloned into the EcoRI and NotI sites of pGEX4T1 (GE Healthcare). All mutations were introduced by QuikChange or QuikChange II Site-Directed Mutagenesis Kits (Stratagene) using complementary mutagenic primer pairs (primer sequences available upon request) into pcDNA1.1/AMP IL2-HT_193_, pGEX4T1-HT_193_, pGEX4T1-hHT_193_, and other m*Pkd1* plasmids [21]. All these mutated sequences (except Q4215P) were subcloned as needed into the pcDNA1.1/AMP or GEX4T1 plasmids using available restriction sites. To produce pGEX4T1-HT_193_ Q4215P, mouse *Pkd1* sequence containing the relevant mutation was PCR-amplified using primers HA-forward and AT-reverse. The product was digested with SacI and NotI, and used to replace the wild-type SacI/NotI fragment of pGEX4T1-HT_193_. All mutations were verified by DNA sequencing. Mutant sequences are shown in Figure S1C. sIg-0 and -HT_193_ construction was described previously [21]. Detailed descriptions of all cloning steps are available upon request.

### Fusion Protein Purification

BL21(DE3) bacteria were grown in LB with 50 µg/ml ampicillin. Overnight cultures were diluted 1∶40 and grown at 37°C, 250 RPM for 1.5 h, then an additional 1.5 h at 20–25°C, 250 RPM before inducing with 0.5 mM IPTG for 2 h at 20–25°C, 250 RPM. Cell pellets were harvested by centrifugation (3,000×*g*, 10 min, 4°C) and stored at −80°C. Cells were lysed in ice cold PBS (140 mM NaCl, 2.7 mM KCl, 10 mM Na_2_HPO_4_, 1.8 mM KH_2_PO_4_, pH 7.4) containing 10% glycerol and 1 mM DTT (PBS wash buffer) in a French Press. Triton X-100 was added to 0.1% and the lysates were clarified twice by centrifugation (12,000×*g*, 15 min, 4°C). Supernatants were then mixed with glutathione (GSH)-Sepharose beads (approximately 0.5 ml per 400 ml bacterial culture) pre-washed in PBS wash buffer with 0.1% Triton X-100, and incubated for 1 h with gentle agitation. Bound proteins were washed four times with 35 ml PBS wash buffer, and fusion proteins were eluted with four 0.5 ml aliquots of 50 mM Tris-HCl, pH 7.5 containing 5 mM reduced glutathione. Glycerol was added to a final concentration of 15% and the fusion proteins were aliquoted and stored at −80°C.

### Cell Culture and Transient Transfection

293T cells (ATCC) were maintained in 5% CO_2_ at 37°C in 1× DMEM with L-glutamine, sodium pyruvate, and 4.5 g/L glucose (Cellgro) supplemented with 10% heat-inactivated fetal calf serum and 10,000 units penicillin/10 mg streptomycin per liter. Cells were transiently transfected by a modified calcium phosphate protocol as described previously [22].

### Immunoprecipitation

Following transient transfection, cells were washed twice in ice-cold DPBS (Cellgro), scraped in IP lysis buffer (150 mM NaCl, 10 mM Tris-HCl pH 7.5, 2 mM EDTA, 1% Triton X-100, 0.5% NP40, 1 mM sodium orthovanadate, 25 mM glycerol-2-phosphate, 25 mM NaF) containing protease inhibitors PMSF (0.2 mM) and 1 µl/ml protease cocktail inhibitor (P8340, Sigma), and rotated for 30 min at 4°C. Lysates were clarified by centrifugation (14,000×*g*, 5 min, 4°C). Protein concentration was determined with the BCA Protein Assay Kit (Pierce), and equivalent amounts of protein were rotated at 4°C with agarose-conjugated HA-probe (F-7; Santa Cruz Biotech) or protein A/G+ agarose pre-washed in IP lysis buffer plus protease inhibitors. After 2 h, bound proteins were washed six times in IP lysis buffer containing 300 mM NaCl, then boiled 4 min in SDS-PAGE sample buffer, resolved by SDS-PAGE, and electrophoretically transferred to PVDF.

### Immunoblotting

PVDF membranes were blocked in TBS/T buffer containing 5% dry milk and 10 mM NaN_3_, then probed with various antibodies diluted in TBS (20 mM Tris-HCl, pH 8.0, 548 mM NaCl) with 0.05% Tween-20, 5% dry milk, and 10 mM NaN_3_. Membranes were washed, then probed with secondary antibodies diluted in TBS with 0.05% Tween-20 and 5% dry milk. Membranes were washed, then incubated 5 min in horseradish peroxidase (HRP) developing buffer (equal parts 131 µM p-coumaric acid, 4.1 mM luminol in 0.1 M Tris-HCl, pH 9.0, and 2% stable peroxide substrate {Pierce}in 0.1 M Tris-HCl, pH 9.0 mixed immediately prior to use) and exposed to film or imaged using a Fluor-S™ MultiImager (BioRad). Anti-human IgG blots were developed as described previously [23]. Antibody dilutions were as follows: PC1 antibody A19 [24] - 1∶10,000; PP1α (C-19; Santa Cruz Biotechnology) - 1∶200; GST (Pharmacia) - 1∶1,000; HA (F-7, Santa Cruz Biotechnology) - 1∶500; Goat Anti-Mouse HRP (BioRad) - 1∶5,000; Goat Anti-Rabbit HRP (BioRad) - 1∶10,000, Rabbit Anti-Goat HRP (Sigma) - 1∶5,000; Goat Anti-human IgG Fc-alkaline phosphatase conjugated antibody (Jackson Immunoresearch) - 1∶10,000. To re-probe, membranes were washed twice for 5 min in 0.2 M NaOH, rinsed in TBS, then re-blocked.

### Protein Phosphorylation and Dephosphorylation

Purified fusion proteins were adsorbed to GSH-Sepharose beads pre-washed in PKA buffer (40 mM Tris-HCl pH 7.4, 20 mM magnesium acetate) and phosphorylated with ∼190 units/ml purified, recombinant catalytic subunit of cAMP-dependent protein kinase (Promega) in PKA buffer with 0.2 mM ATP and 50 µCi/ml [γ-^32^P]ATP, 3,000 Ci/mmol (PerkinElmer). Bound, radio-labeled proteins were washed twice in ice-cold PP1 or PP2B buffer (PP1∶50 mM HEPES, pH 7.2, 10 mM MgCl_2_, 1 mM MnCl_2_, 1 mM DTT, 0.1% BSA; PP2B: 50 mM Tris-HCl, pH 7.4, 1 mM NiCl_2_, 0.05% BSA) containing 2 mM ATP, twice in ice-cold phosphatase buffer, then incubated with ∼1.8 units/ml purified, recombinant PP1α (Millipore) or PP2B (Promega) and 10 µg/ml calmodulin (Upstate). Reactions were terminated by the addition of 6× SDS-PAGE sample buffer and held on dry ice until being boiled 4 min and resolved by SDS-PAGE. Proteins were electrophoretically transferred to PVDF, and ^32^P incorporation was detected by autoradiography or phosphorimaging using a PhosphorImager SI (Molecular Dynamics) and Typhoon 9410 Variable Mode Imager (Amersham Biosciences).

### Quantification

Protein band intensity was quantified following immunoblotting and ^32^P incorporation was quantified following phosphorimaging using Quantity One software version 4.2.1 (BioRad).

### Statistics

Data are means ± SE. Unless noted otherwise, statistical significance was determined by one-way ANOVA and Student-Newman-Keuls (S-N-K) posttest for multiple comparisons or unpaired t-test for comparisons between control and experimental groups.

## Results

### PC1 interacts with PP1α

Sequence analysis of the C-terminal cytosolic tail of mouse PC1 revealed the presence of three highly conserved putative PP1-binding motifs (RVxF or FxxBxB) [1], [6] (Figure 1A, boxed residues and up arrows). These sequences lie in a 23-amino acid region that is within 15 amino acids of a conserved serine residue (asterisk at S4159) that we had previously determined to be the major site of PKA phosphorylation in the C-terminal tail of mouse PC1 [24]. The putative PP1-binding motifs lie in the most highly conserved region of the protein [17] and overlap with the G protein activation domain as well as a region thought to function as a nuclear localization signal for a C-tail proteolytic cleavage fragment [25]. The high conservation of this region suggests that it is vital for PC1 function, which is supported by the presence of disease-associated mutations in this region of the protein [26].

**Figure 2:**
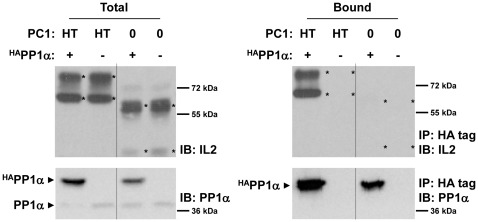
PC1 interacts with PP1α. To determine whether PC1 can interact with PP1, 293T cells were transfected with plasmids encoding hemeagglutin (HA) epitope-tagged PP1α (^HA^PP1α) and the C-terminal, cytosolic 193 amino acids of PC1 fused to the extracellular and transmembrane portion of the IL2 receptor (IL2-HT_193_). Empty plasmid and an IL2 construct lacking PC1 sequence (IL2-0) were used as controls for ^HA^PP1α and IL2-HT_193_, respectively. Lysates from the transfected cells were immunoprecipitated (IP) with anti-HA antibodies to pull down PP1α. Antibody-bound and total fractions were resolved by SDS-PAGE and immunoblotted (IB) with anti-IL2 antibodies. Blots were then stripped and re-probed with anti-PP1α antibody. All IL2 and sIg fusion proteins (including IL2-0 and sIg-0) used in this study migrate as doublets, presumably due to a modification of the IL2- and sIg-portions of the fusion proteins. Asterisks are used to mark the IL2- and sIg-specific bands (or their positions were they to be present). Solid arrows are used to indicate the position of other proteins. Different parts of this same gel image (as well as other gel images represented in this manuscript) have been cut and rearranged for consistency and clarity. All other modifications, such as resizing or adjustments to contrast, are performed such that all groupings of images from different parts of the same gel are treated identically. Dashed lines indicate image borders that have been spliced together.

To determine whether PC1 can interact with PP1, 293T cells were transfected with plasmids encoding HA epitope-tagged PP1α and the C-terminal, cytosolic 193 amino acids of mouse PC1 fused to the extracellular and transmembrane portion of the IL2 receptor (IL2-HT_193_, Figure 1B). Cell lysates were immunoprecipitated with anti-HA antibodies, and co-precipitating proteins were detected by SDS-PAGE and immunoblotting. As can be seen in Figure 2, ^HA^PP1α readily co-precipitated the PC1 IL2-HT_193_ protein but not the IL2 control protein (IL2-0). Reciprocal co-immunoprecipitation was demonstrated (see Figure S2) using ^HA^PP1α and an identical region of the PC1 C-tail fused to the membrane targeting cassette, sIg.7 [22].

### Analysis of PP1α Binding Determinants

**Figure 3:**
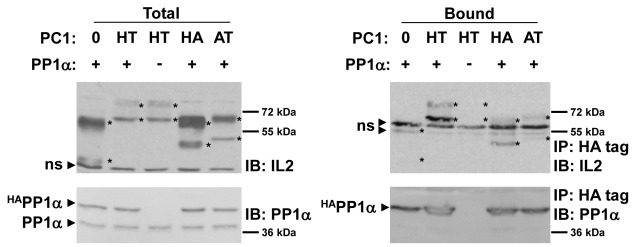
Immunoprecipitation of PC1 truncation proteins. To determine the region of PC1 responsible for interacting with PP1α, various IL2-PC1 fusion proteins were tested for their ability to co-immunoprecipitate with PP1α. The HA_74_ region of PC1 contains the putative PP1-binding motif, but lacks the coiled-coil domain. The AT_120_ region of PC1 lacks the putative PP1-binding motif, but contains the coiled-coil domain. Cell lysates from transfected 293T cells were immunoprecipitated and immunoblotted as described in Figure 2. Additional replicates of this experiment can be seen in Figures S3. ns  =  non-specific band.

To determine the region of PC1 responsible for interacting with PP1α, IL2-PC1 fusion proteins (Figure 1B) encoding either the membrane distal 120 amino acids (IL2-AT_120_, a region which lacks the putative PP1-binding and PKA phosphorylation sites but contains the coiled-coil) or the membrane proximal 74 amino acids (IL2-HA_74_, a region which lacks the coiled-coil but contains the putative PP1-binding and PKA phosphorylation sites) of the C-terminal tail of PC1 were tested for their ability to co-immunoprecipitate with PP1α. As shown in Figure 3 (also Figure S3), both of these truncations (IL2-AT_120_ and -HA_74_) dramatically reduced, but failed to eliminate binding of PP1α to PC1, as compared to the longer IL2-HT_193_ protein. No binding of IL2-AT_120_ and HA_74_ occurred in the absence of co-expressed PP1α (data not shown). Weak non-specific binding of IL2-0 to PP1α could only be detected upon over-expression of IL2-0 (see Figure S3). These results suggest that multiple PC1 sites are involved in PP1α binding and that PP1α interacts with the conserved PP1-binding motif plus additional elements within the membrane distal portion of the PC1 C-tail. It is also possible that additional PC1-interacting proteins may be required to stabilize the interaction.

### PKA-phosphorylated PC1 is a PP1α Substrate

**Figure 4:**
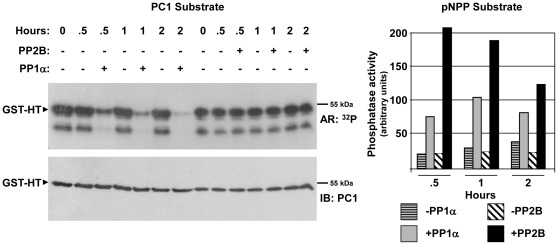
Dephosphorylation of PC1 by PP1α. To determine whether PP1α can dephosphorylate PKA-phosphorylated PC1, a GST-PC1 C-tail fusion protein (GST-HT_193_) was purified from bacteria, bound to GSH-agarose beads, and phosphorylated by PKA with [γ-^32^P]ATP. Unincorporated ^32^P was removed by washing the fusion protein with an excess of cold ATP in PP1α or PP2B reaction buffer. The radiolabeled protein was then incubated in the presence or absence of purified, recombinant PP1α or PP2B (calcineurin) for 0–2 h. Aliquots of the reaction were removed and tested for phosphatase activity against the generic substrate p-nitrophenyl phosphate (pNPP) or frozen on dry ice in 1× SDS-PAGE sample buffer to terminate the phosphatase reaction. Terminated reactions were resolved by SDS-PAGE, and phosphorylated fusion protein was detected first by autoradiography (AR) followed by immunoblotting.

Based on the proximity of the putative PP1-binding motifs to the S4159 PKA phosphorylation site [24], we hypothesized that PP1α might be able to specifically dephosphorylate PKA-phosphorylated PC1. To test this hypothesis, the GST-HT_193_ fusion protein (see Figure 1B) was purified from bacteria and phosphorylated by PKA with [γ-^32^P]ATP. The radiolabeled protein was incubated with purified, recombinant PP1α or PP2B (calcineurin) in an *in vitro* phosphatase assay. Following incubation, the proteins were electrophoresed and detected by autoradiography and immunoblotting. PC1 phosphorylation was stable over a 2 h assay period in the absence of phosphatase. Both phosphatases were able to dephosphorylate the generic phosphatase substrate p-nitrophenyl phosphate (pNPP). However, only PP1α was able to dephosphorylate PC1 (Figure 4), suggesting that dephosphorylation of PC1 by PP1α may be specific.

### Human PC1 is Dephosphorylated by PP1α

**Figure 5:**
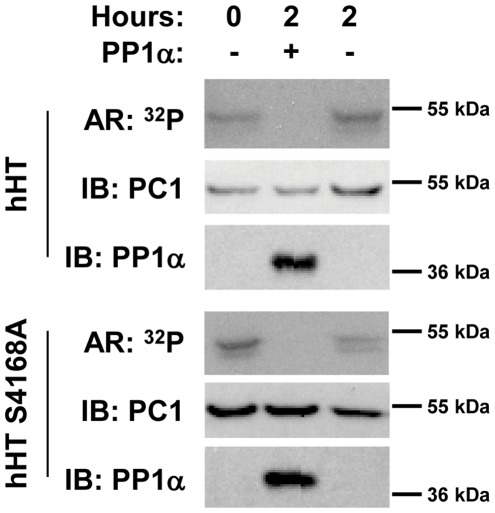
Dephosphorylation of human PC1 by PP1α. To determine whether PP1α can also dephosphorylate PKA-phosphorylated human PC1 (hPC1), GST-hPC1 C-tail fusion proteins (GST-hHT_193_ and GST-hHT_193_ S4168A) were purified, phosphorylated, and detected as described in Figure 4. GST-hHT_193_ S4168A was used in this analysis to be certain that phosphorylation and dephosphorylation was occurring on a residue other than S4168, which is equivalent to the site of PKA phosphorylation on mouse PC1 (S4159).

In contrast to mouse PC1, which is phosphorylated by PKA on S4159, human PC1 is phosphorylated by PKA at a different position on a non-conserved serine [27]. To determine whether PP1α dephosphorylates PKA-phosphorylated human PC1, a GST fusion protein containing the C-terminal 193 amino acid residues of human PC1 (GST-hHT_193_, Figure 1B) was tested in the *in vitro* phosphatase assay. As shown in Figure 5, the GST-hHT_193_ fusion protein was readily dephosphorylated by PP1α. A fusion protein in which hS4168 (equivalent to the S4159 PKA phosphorylation site of mouse PC1) was mutated to alanine was also readily phosphorylated and dephosphorylated. These results confirm that PKA phosphorylation occurs on distinct residues at different locations in mouse and human PC1 but that both mouse and human PC1 are dephosphorylated by PP1α.

### Mutation of RVxF Residues Interferes with PC1 Dephosphorylation

**Figure 6:**
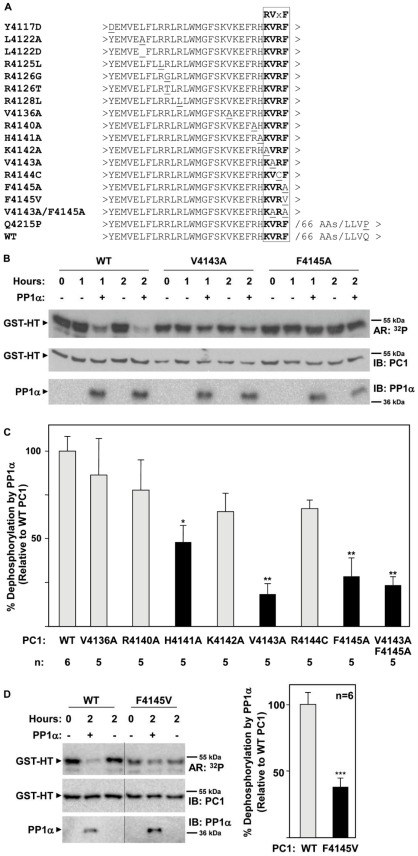
Mutations within the RVxF motif prevent dephosphorylation of PC1 by PP1α. To determine whether mutations in the RVxF motif affect the ability of PP1α to dephosphorylate PC1, GST-HT_193_ fusion proteins with point mutations within and around this motif and GST-HA_74_ were analyzed in an *in vitro* kinase/phosphatase assay as described in Figure 4. The amount of phosphorylated protein (relative to input material at the start of the reaction) remaining after 2 h in the presence or absence of PP1α was determined by autoradiography and immunoblotting. Following detection of the fusion proteins, membranes were stripped and re-probed for the presence of PP1α. (A) Sequence of wild type (WT), and mutant RVxF constructs of PC1. The primary amino acid sequence spanning the various sites of mutagenesis is shown. The identity of the mutated residue is shown at left and underlined in the primary sequence. The RVxF motif is in bold. (B) Representative autoradiographs and immunoblots showing phosphorylation and total protein levels of GST-HT_193_ WT, V4143A, and F4145A PC1 fusion proteins. (C) Summary of the effects of PC1 mutations on PP1α-mediated dephosphorylation of PC1. On average, approximately 67% of WT input material was dephosphorylated over the 2 h assay period. Data (mean ± SE) represent the percent dephosphorylation of PC1 constructs by PP1α, relative to dephosphorylation of WT PC1 (set to 100%). n = 6 for WT and n = 5 for mutant constructs. *P<0.05 and **P<0.01, compared to the effect of PP1α on WT PC1, determined by one-way ANOVA and the Dunnett multiple comparison post-test. Representative autoradiographs and immunoblots for GST-HT_193_ V4136A, R4140A, H4141A, K4142A, R4144C, and V4143A/F4145A are shown in Figure S4A. Representative autoradiographs and immunoblots for additional mutants which lacked obvious defects in PP1α dephosphorylation are shown in Figure S4B. (D) Summary and representative autoradiographs and immunoblots showing phosphorylation and total protein levels of GST-HT_193_ WT and F4145V PC1 fusion proteins. Data (mean ± SE) represent the percent dephosphorylation of PC1 constructs by PP1α, relative to dephosphorylation of WT PC1 (set to 100%). n = 6 for WT and F4145V. ***P<0.001, compared to the effect of PP1α on WT PC1, determined by unpaired t test.

Mutation of the critical hydrophobic residues in the RVxF PP1-binding motif to alanine frequently disrupts the interaction between the phosphatase and its regulatory proteins [5], [28], [29], [30]. To examine this, we tested whether a series of mutations (Figure 6A), including V4143A and F4145A in the RVxF motif, would disrupt the ability of PP1α to dephosphorylate PC1 using GST-HT_193_ fusion proteins containing these mutations in an *in vitro* phosphatase assay. As seen in Figure 6B, V4143A and F4145A mutations dramatically blocked the ability of PP1α to dephosphorylate PC1, thus indicating the importance of the RVxF motif in PP1α function. To determine if other residues in and around the RVxF motif also play a critical role, those residues in close proximity to the RVxF motif were mutated and tested (Figure 6C). These included R4140A, H4141A, K4142A, V4143A/F4145A, and R4144C. The R4144C mutation corresponds to a human disease-associated mutation in the “x” residue of the RVxF motif [31]. As a control, a V4136A mutation was also tested. V4136 lies in close proximity to the RVxF motif and within an amino acid sequence that is similar to but does not conform to an RVxF motif (KVKEF; V4136 underlined).

The double mutant V4143A/F4145A blocked dephosphorylation of PC1 to an extent comparable to that of single mutants V4143A and F4145A (Figure 6C). Mutations V4136A, R4140A, K4142A, and R4144C did not have a significant effect on dephosphorylation of PC1. However, mutation H4141A did have a pronounced effect on the ability of PP1α to dephosphorylate PC1, although not to the same extent as V4143A and F4145A. Representative autoradiographs and immunoblots of each mutant are shown in Figures 6B or S4A. These results demonstrate that mutagenesis of the critical hydrophobic residues (V4143A and F4145A) in the RVxF motif, as well as the residue in the −1 position (H4141A), interferes with the ability of PP1α to dephosphorylate PC1.

A number of additional mutants, some representing naturally-occurring human polymorphisms, were tested but did not appear to affect dephosphorylation by PP1α, suggesting that the RVxF motif is highly specific for PP1α function. Several of these additional mutations were located in or near the upstream FxxBxB motif, suggesting that these residues do not function as critical determinants of the ability of PP1α to dephosphorylate PC1. Representative autoradiographs and immunoblots of these additional mutant proteins can be seen in Figure S4B.

We also tested the ability of ADPKD-associated mutation F4145V to disrupt dephosphorylation of PC1. This mutation, which affects the critical phenylalanine in the PC1 RVxF motif, was predicted to be “probably pathogenic” by multiple sequence analysis tools and segregated with ADPKD individuals [32]. As seen in Figure 6D, this mutation also significantly reduced dephosphorylation of PC1. This observation suggests that the PC1/PP1 interaction and the phosphorylation of PC1 may be clinically relevant.

### Mutation of RVxF Residues does not Disrupt PC1-PP1α Binding *in vivo*

Having determined that mutation of the critical hydrophobic residues in the RVxF motif blocks dephosphorylation of PC1, we next asked whether these mutations disrupt the binding interaction between PC1 and PP1α *in vivo*. To examine this, IL2-HT_193_ fusion proteins containing various RVxF mutations were tested for their ability to co-immunoprecipitate with PP1α from lysates of co-transfected 293T cells. None of the mutations were found to affect the ability of PP1α to co-immunoprecipitate PC1 (see Figure S5). These results support the idea that the interaction between PP1α and PC1 involves multiple binding elements, and suggest that mutations in the critical hydrophobic residues of the PC1 RVxF motif disrupts the ability of PP1α to dephosphorylate PC1 possibly without significantly disrupting binding.

## Discussion

We have identified a putative PP1-binding motif (RVxF) in the C-terminal, cytosolic tail of PC1 and demonstrated that PC1 interacts with and is dephosphorylated by PP1α. We have also identified mutations in the putative PP1-binding motif of PC1 that interfere with PP1α-mediated dephosphorylation of PC1. These results are the first to demonstrate an interaction between PC1 and a serine/threonine phosphatase and the first to identify the phosphatase responsible for dephosphorylating PKA-phosphorylated PC1. Recently, PC1 was also shown to interact with and be dephosphorylated by a member of the LAR-family of tyrosine phosphatases [33]. While our studies have focused on PP1α, we cannot rule out the possibility that PC1 interacts with other PP1 isoforms. Many PP1 regulatory proteins do, in fact, bind to all isoforms of PP1 [34], and we have observed binding of PC1 to the β as well as the α isoform of PP1 (data not shown).

An interaction between PC1 and PP1α was hypothesized based on the presence of a highly conserved RVxF motif in the C-terminal, cytosolic tail of PC1 (Figure 1A). The interaction was confirmed by co-immunoprecipitation of fusion proteins transiently expressed in mammalian cells (Figure 2, S2). Deletion of the putative PP1-binding domain dramatically reduced binding (Figures 3, S3; AT_120_ construct). A C-terminal truncation that left the PP1-binding motif intact also diminished binding to a similar extent (Figures 3, S3; HA_74_ construct). However, neither of these truncations failed to completely eliminate binding. We also observed that RVxF mutations that interfered with dephosphorylation of PC1 did not significantly diminish the ability of PC1 to be co-immunoprecipitated with PP1α (Figure S5).

**Figure 7:**
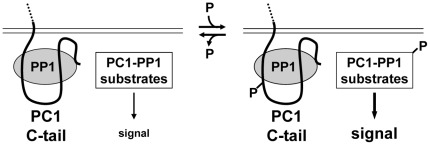
Model of PC1-PP1α holoenzyme function. In this model we speculate that PP1 regulates the phosphorylation status of PC1 and PC1-interacting proteins, and that disruption of the PC1-PP1 holoenzyme complex may lead to altered signaling and cystogenesis due to misregulation of protein phosphorylation.

A possible explanation for these observations is that PC1 contains multiple PP1-binding determinants. In some cases, mutation of critical residues within the RVxF domain of known PP1-regulatory proteins can completely disrupt binding [30]. In other cases, these mutations have no obvious effect on binding to PP1 when expressed in the context of a large polypeptide or full-length protein, but can disrupt binding between PP1 and interacting short peptide regions derived from the full-length protein [6], [29]. Thus, PP1 regulatory proteins are likely to have multiple sites of interaction that facilitate stable interactions and help dictate the unique substrate specificity for a given PP1 holoenzyme [34], [35]. Consistent with this, other PP1-interacting motifs have been identified, including the myosin phosphatase N-terminal element (MyPhoNE) [8], [36], the SILK motif [8], [37], [38], [39], and the FxxBxB motif [6], [7]. Of these, the MyPhoNE and SILK motifs have been identified in many PP1-regulatory proteins, although they are not present in PC1. PC1 does contain two FxxBxB motifs in close proximity to the RVxF motif. However, mutations that affected these residues did not disrupt dephosphorylation. Furthermore, these motifs are localized in the IL2-HA_74_ protein, which binds to PP1α poorly. While we cannot rule out contributions of the FxxBxB motifs to the PC1-PP1α interaction, it seems likely that if additional binding determinants are present in the C-tail of PC1, they reside distal to the RVxF motif in the C-terminal 120 amino acids.

Another potential explanation is that the interaction between PC1 and PP1α is not direct. Numerous binding partners of PC1 have been identified, with a number of interactions occurring via the PC1 coiled-coil domain [40]. Given that deletion of the region of PC1 containing the coiled-coil dramatically reduced binding to PP1α, it seems possible that additional proteins that interact via the PC1 coiled-coil might stabilize the interaction between PC1 and PP1α.

The ability of PC1 to interact with PP1α raised the possibility that PP1α may be the phosphatase responsible for dephosphorylating PKA-phosphorylated PC1. We confirmed that PP1α dephosphorylates PKA-phosphorylated PC1 in an *in vitro* phosphatase reaction (Figure 4). This reaction appears to be specific for PP1α, since PP2B was incapable of dephosphorylating PC1, despite having higher activity toward a generic substrate. Furthermore, mutation of critical hydrophobic residues (V4143 and F4145) in the RVxF PP1-binding motif and H4141 in the -1 position relative to the motif disrupted dephosphorylation of PC1 (Figure 6). Thus, PC1 appears not only to be a PP1 regulatory protein, but also a PP1 substrate. An ADPKD-associated mutation affecting F4145 (F4145V) also disrupted dephosphorylation of PC1, suggesting that the PC1/PP1 interaction and the phosphorylation of PC1 may be clinically relevant. Interestingly, both human and mouse PC1 are substrates for PP1α (Figure 5), despite the fact that PKA phosphorylation occurs at different sites in the two proteins.

The ability of the H4141A mutation to interfere with the dephosphorylation of PC1 (Figure 6) is consistent with a recently published detailed analysis of the RVxF motif and surrounding amino acids [8]. This analysis of over 140 validated PP1-interacting proteins suggests that the binding motif should be redefined as RRVxF to reflect the prevalence of a basic residue in the -1 position relative to the currently accepted RVxF motif. While histidine is under-represented at this position in the compiled sequence of validated PP1-interacting proteins, histidine does have the potential to behave as a basic residue. Furthermore, H4141 of PC1 lies in a cluster of basic residues which includes K4135, K4137, R4140, and K4142. Interestingly, amongst the PP1-interacting proteins analyzed, there seems to be some tolerance for alanine in the second but not the first basic residue position of the expanded RRVxF binding motif (RAVxF, prevalence of ∼7.5% vs. ARVxF, never represented). Consistent with this observation, mutation H4141A (ARVxF) but not K4142A (RAVxF) had a statistically significant effect on PP1α-mediated dephosphorylation of PC1. Our data would suggest that the presence of a basic residue in the first basic position is more important than its presence in the second. Also of interest, PC1 contains lysine and arginine residues in the –4 and –1 positions of the expanded RRVxF motif, respectively, which are the residues most frequently represented at these positions in the compiled sequence of validated PP1-interacting proteins [8]. Thus, the amino acid sequence in and around the putative PP1-binding motif of PC1 is actually very similar to the amino acid sequence of PP1-binding domains found in a large number of validated PP1-regulatory proteins.

The proximity of the PKA phosphorylation site to the PP1-binding motif is intriguing. Perturbation of the sites of interaction between PP1 and its regulatory proteins by changes such as phosphorylation can dramatically alter the activity of the holoenzyme [35], [41], [42], [43]. For example, the PP1-regulatory proteins dopamine- and cAMP-regulated phosphoprotein with molecular weight 32 kDa (DARPP-32) and inhibitor-1 are both inhibitors of PP1 activity. These regulatory proteins bind PP1 equally well regardless of their phosphorylation state, but are poor inhibitors in the unphosphorylated state. Upon phosphorylation, the IC_50_ of these inhibitors increases by 1,000 fold, presumably by forming a pseudo-substrate complex with the phosphatase [1]. In contrast, the negative regulation of PP1 exerted by another PP1 regulatory protein, nuclear inhibitor of protein phosphatase 1 (NIPP1), is relieved by PKA and casein kinase 2 phosphorylation of NIPP1 [30].

PP1-regulatory proteins that are also substrates can integrate competing signals from the phosphorylating kinase and PP1. A prime example of this mode of regulation is that of the kinase Nek2, a PP1-regulatory protein that induces centrosome splitting. Nek2 is activated by autophosphorylation, and is thought to trigger centrosome splitting by phosphorylating cohesion proteins such as C-Nap1 [44]. Nek2 and C-Nap1 are likely dephosphorylated by associated PP1, whose activity is modified by ATM and CDKs [2]. Thus, centrosome splitting appears to be controlled by multiple inputs converging on the ability of the PP1-regulatory protein and substrate Nek2 to remain phosphorylated. We speculate that a PC1-PP1α holoenzyme complex may function in a similar manner.

In Figure 7, we present a model of PC1-PP1 holoenzyme function whereby signaling events downstream of PC1 are regulated by the competing actions of PP1 and cellular kinases acting on PC1 itself or other, putative substrates of the holoenzyme. For example, PP1 may serve as a molecular switch that antagonizes PC1-mediated signaling initiated by events such as PKA-mediated phosphorylation of PC1. Alternatively, PP1 may regulate the phosphorylation of PC1-interacting proteins such as PC2, a protein whose localization and activation are known to be affected by phosphorylation [45], [46], [47], [48]. In our model, we predict that perturbation of PC1-PP1 holoenzyme function, either by complete loss of PC1 or by PC1 mutations that affect the activity of PP1 in complex with PC1 (*e.g.* RVxF mutations, Figure 6), would lead to disregulated phosphorylation and cell signaling that could potentially initiate or exacerbate cystogenesis. In summary, we have identified PC1 both as a regulatory subunit and as a substrate for PP1α. We propose that PC1 and PP1α form a holoenzyme complex in which the two proteins stably interact and are poised to dephosphorylate substrates required for normal cellular functions. The identification of PC1 mutations that interfere with the activity of PP1α in complex with PC1 should facilitate efforts to understand the role of PP1α in PC1-mediated signaling as well as the role of PKA phosphorylation in the function of PC1.

## Supporting Information

Figure S1 **Pkd1 construct and primer sequences.** (A) Coding and 3′ non-coding sequence of pcDNA1.1/AMP IL2-HT_193_: The ATG start codon is at the 5′ end followed by IL2 sequence. The polycystin-1 (PC1) encoding region of IL2-HT_193_ is in bold. Vector sequences are in lower case. EcoRI (GAATTC) and NotI (GCGGCCGC) restriction sites are underlined. (B) PCR primer sequences: *the AT reverse primer is complementary to both human *PKD1* and mouse *Pkd1*; **the bold underlined base in primer sequence human (h) HT-forward introduces a silent mutation into the hHT_193_ sequence. This mutation was necessary to disrupt secondary structure in the original primer sequence, which prevented efficient priming. (C) Sequence of mutant *PKD1* clones: The sites of mutation within mouse *Pkd1* and human (h) *PKD1* are shown. Mutated residues are in bold; affected codons are underlined.
(DOCX)

Figure S2 **PP1α immunoprecipitates with PC1.** 293T cells were transfected with plasmids encoding hemeagglutin (HA) epitope-tagged PP1α (^HA^PP1α) and the C-terminal, cytosolic 193 amino acids of PC1 fused to the membrane targeting cassette, sIg.7 [22]. Empty plasmid and a sIg construct lacking PC1 sequence (sIg-0) were used as controls for ^HA^PP1α and sIg-HT_193_, respectively. Lysates from the transfected cells were immunoprecipitated (IP) with protein A/G+ agarose to pull down sIg- constructs. Bound and total fractions were resolved by SDS-PAGE and immunoblotted (IB) with anti-HA antibodies to detect ^HA^PP1α, then stripped and re-probed with anti-human IgG Fc-alkaline phosphatase conjugated antibody to detect sIg-constructs. While some ^HA^PP1α could be detected in the sIg-0 bound fractions, we consistently observed enrichment of ^HA^PP1α when precipitated with sIg-HT_193_.
(TIF)

Figure S3 **PC1 immunoprecipitates with PP1α.** Panels A, B, and C are replicate co-immunoprecipitation experiments (see Figure 2). Note in A and B that there is less PP1α present in the IL2-HT_193_ bound fraction as compared to all other lanes, and yet there is significantly more IL2-HT_193_ co-immunoprecipitated than IL2-HA_74_, -AT_120_, or −0. Also note that more IL2-HT_193_ can be detected in the bound fraction of B despite dramatic over-expression of IL2-HA_74_ as demonstrated in the total fraction. In A and C, some IL2-0 can be detected in the bound fraction. However, the amount of IL2-0 in the bound fraction is never greater than that of any PC1 protein, and the amount of IL2-0 present in the total fraction is significantly greater than the amount of any PC1 protein. No IL2-0 was detected in the bound fraction of B. ns  =  non-specific band.
(TIF)

Figure S4 **Representative autoradiographs and immunoblots of** ***in vitro*** **kinase/phosphatase assays.** (A) Analysis of PC1 mutants V4136A, R4140A, H4141A, K4142A, R4144C, and the double mutant V4143A/F4145A was conducted as described in Figure 5. (B) Additional PC1 mutants as well as PC1 truncation protein HA_74_ were analyzed by autoradiography and immunoblotting as described in Figure 5. Mutations L4122Δ, R4125L, R4126G, R4126T, and R4128L are either naturally occurring PKD associated mutations and/or lie in or near the upstream FxxBxB site in PC1 (Figure 1A) [6], [7], [31], [49]. In addition, we had previously demonstrated that mutations L4122Δ, R4126G, and R4126T inhibit heterotrimeric G protein signaling (see Magenheimer, B. S., et al. (2002) *J Am Soc Nephrol* **13**, 18A F-FC085). Q4215P is a PKD associated mutation that interrupts the coiled-coil domain of PC1 [50]. Y4117D was examined as it is a potential site of tyrosine phosphorylation [17]. GST-HA_74_ was examined because IL2-HA_74_ co-immunoprecipitates poorly with PP1α from lysates of co-transfected 293T cells (Figure 3).
(TIF)

Figure S5 **RVxF mutations fail to disrupt the PC1-PP1α interaction.** To determine whether RVxF mutations disrupt binding between PC1 and PP1, 293T cells were transfected and immunoprecipitated as described in Figure 2 but with wild type and RVxF-mutated IL2-HT_193_ fusion proteins. RVxF mutations V4143A and F4145A failed to appreciably decrease binding with ^HA^PP1α.
(TIF)
