# Supplementary material for: Protein Phosphatase-1α Interacts with and Dephosphorylates Polycystin-1
Source: PLoS One. 2012 Jun 4;7(6):e36798. doi: 10.1371/journal.pone.0036798 (PMC3366979; doi:10.1371/journal.pone.0036798)
Supplement: Figure S1 — Pkd1 construct and primer sequences. (A) Coding and 3′ non-coding sequence of pcDNA1.1/AMP IL2-HT193: The ATG start codon is at the 5′ end followed by IL2 sequence. The polycystin-1 (PC1) encoding region of IL2-HT193 is in bold. Vector sequences are in lower case. EcoRI (GAATTC) and NotI (GCGGCCGC) restriction sites are underlined. (B) PCR primer sequences: *the AT reverse primer is complementary to both human PKD1 and mouse Pkd1; **the bold underlined base in primer sequence human (h) HT-forward introduces a silent mutation into the hHT193 sequence. This mutation was necessary to disrupt secondary structure in the original primer sequence, which prevented efficient priming. (C) Sequence of mutant PKD1 clones: The sites of mutation within mouse Pkd1 and human (h) PKD1 are shown. Mutated residues are in bold; affected codons are underlined. (DOCX) [file pone.0036798.s001.docx]

**A**

ATGGATTCATACCTGCTGATGTGGGGACTGCTCACGTTCATCATGGTGCCTGGCTGCCAGGCAGAGCTCTGTGACGATGACCCGCCAGAGATCCCACACGCCACATTCAAAGCCATGGCCTACAAGGAAGGAACCATGTTGAACTGTGAATGCAAGAGAGGTTTCCGCAGAATAAAAAGCGGGTCACTCTATATGCTCTGTACAGGAAACTCTAGCCACTCGTCCTGGGACAACCAATGTCAATGCACAAGCTCTGCCACTCGGAACACAACGAAACAAGTGACACCTCAACCTGAAGAACAGAAAGAAAGGAAAACCACAGAAATGCAAAGTCCAATGCAGCCAGTGGACCAAGCGAGCCTTCCAGGTCACTGCAGGGAACCTCCACCATGGGAAAATGAAGCCACAGAGAGAATTTATCATTTCGTGGTGGGGCAGATGGTTTATTATCAGTGCGTCCAGGGATACAGGGCTCTACACAGAGGTCCTGCTGAGAGCGTCTGCAAAATGACCCACGGGAAGACAAGGTGGACCCAGCCCCAGCTCATATGCACAGGTGAAATGGAGACCAGTCAGTTTCCAGGTGAAGAGAAGCCTCAGGCAAGCCCCGAAGGCCGTCCTGAGAGTGAGACTTCCTGCCTCGTCACAACAACAGATTTTCAAATACAGACAGAAATGGCTGCAACCATGGAGACGTCCATATTTACAACAGATCTCCAGGTAGCAGTGGCCGGCTGTGTTTTCCTGCTGATCAGCGTCCTCCTCCTGAGTGGGCTCACCTGGCAGCGGAGACAGAGGAAGTCTAGAATTCCC**CACGCCTTGCGTGGCGAGCTCTACCGCCCAGCATGGGAACCCCAGGACTATGAGATGGTGGAGCTTTTCCTGCGTAGGCTTCGGCTCTGGATGGGCTTCAGCAAGGTCAAGGAGTTCCGCCACAAAGTCCGCTTTGAAGGAATGGATCCACTGCCTTCCCGCTCATCCAGGGGCTCCAAGTCATCCCCAGTTGTGCTCCCACCTAGCTCAGGCTCAGAAGCTTCACACCCATCCACCTCGTCCAGCCAACCAGACGGGCCAAGCGCCAGCTTAAGCCGCTCGACGCTGAAGCTGGAACCAGAGCCCTCTCGCCTCCATGCTGTGTTTGAAAGTCTGCTTGTCCAGTTTGACCGACTCAACCAGGCCACAGAGGACGTCTACCAGCTGGAGCAACAACTCCAGAGCCTTCGAGGCCATGGGCACAATGGACCTCCTTCCTCTCCCTCCCCTGGCTGCTTCCCAGGCTCTCAGCCAGCTTTGCCCAGCCGCCTTTCTCGGGCCAGTCAGGGGCTGGATCAGACTGTAGGCCCCAACAGGGTGTCCCTGTGGCCTAATAACAAGGTCCACCCCAGCAGCACT**TAGGCCCTAGGGGTCTTGGCCATTCCCTTCCCTGGGAATGCCTGAGCTTCACACTGGCCTCTCAGAGCCAGGGTGGACACCACTCAGTATTACCTTCTGCTGCCCTCTAGGTTGGGCCAGGCAGAACGGCTGCATGCCAGTTCTTTTGGGTACAGGTATTGCTGCCTTCCTTACCTGTCCACATATGGGGCTTCTGCACTTTAAAAAGGCTGTGTGGCCAGCCAGGACCCAGGGTCCCCTCCCCACAGGAGGACACAGCAGTATTGGACCAAGTGAGTACCCAGCCTCCAAGACGCTAATTTATTCGCCCCCCCTCCCCCCAAGTCCTCAGGTTCAGCGGGCTGCGCCCAGCTCTACCCCTAGGTGGCCATCTCCTCTTGCTAAAAATCTAAGCTGGAGGGAGGGCTAAGCTACACTTCTCCTTTATCCGTCCCCTAAGTTATTACCTCTCTTGTTTCTGCAGCATACTTGCCCTCTAGCTACCATCTGCTTTCTATGTCCACCATCAATAATTTATATGGGGTTAAAATGTATATATTTTTGTATGTCAGTATTTTCTACTTGGGCTGAAAATGGGTCCATTGTTACCCAGGGCAGGGGTACAGGGGAAGCTGCAGCTATTACTGGATCCGAGTCTGGCCTCAGGCAGGTGCTGGACAGCAATTGGGGCCACTGTGGCCTTGTTTTCCTCCCTTGTCTGGGGCCAGGCAGCACGCAGATCTGCTGGTTTCAGATCTGAGCAAGGGCAGGACTACTGTGGGATGAAAGATGCCAAGAAGTTACTGGACAAGCCCACCAATGGGGCTTGCCCCCATGGCGGGTGGGGGAAAGAGAATGTATGTGATGGCACCTGCCTGCTTCTGTGGGCTCATCTGCAGCTCTAGCCTGGATTCTGCCCCAACCCCAAACAGACAAGACAAAGTCAAATAAAGAAGCTGTCTGACaaaaaaaagcttgggggatccgtcgactcgagcggccgc

**B**

HA-forward 5’ GAGGACCGCGAATTCCCCACG 3’

HA-reverse 5’ GATGCGGCCGCTAAGCTTCTGAGCCTGAGCTAG 3’

AT-forward 5’ GATGAATTCCAGCTTCACACCCATCCACCTC 3’

AT-reverse* 5’ GATGCGGCCGCCTAAGTGCTGCTGGGGTGGAC 3’

hHT-forward** 5’ GATGAATTCCA**T**GCCTTGCGTGGAGAGCTGTAC 3’

**C**

Y4117D GAC**G**ATGAG

WT GACTATGAG

L4122A GAG**GCC**TTC

L4122Δ GAG___TTC

WT GAGCTTTTC

R4125L CTGC**T**TAGGCTTCGGCTC

R4126G CTGCGT**G**GGCTTCGGCTC

R4126T CTGCGTACGCTTCGGCTC

R4128L CTGCGTAGGCTTTGGCTC

WT CTGCGTAGGCTTCGGCTC

V4136A AAGG**C**CAAG

WT AAGGTCAAG

R4140A TTC**GC**CCACAAAGTCCGCTTTGAA

H4141A TTCCGC**GC**CAAAGTCCGCTTTGAA

K4142A TTCCGCCAC**GC**AGTCCGCTTTGAA

V4143A TTCCGCCACAAAG**CG**CGCTTTGAA

R4144C TTCCGCCACAAAGTC**T**GCTTTGAA

F4145A TTCCGCCACAAAGT**G**CGC**GC**TGAA

F4145V TTCCGCCACAAAGTCCGC**GT**TGAA

V4143A/F4145A TTCCGCCACAAAG**CG**CGC**GC**TGAA

WT TTCCGCCACAAAGTCCGCTTTGAA

Q4215P GTCC**C**GTTT

WT GTCCAGTTT

hS4168A GGC**G**CCAAG

hWT GGCTCCAAG
