# Supplementary figures and images for: Protein Phosphatase-1α Interacts with and Dephosphorylates Polycystin-1
Source: PLoS One. 2012 Jun 4;7(6):e36798. doi: 10.1371/journal.pone.0036798 (PMC3366979; doi:10.1371/journal.pone.0036798)

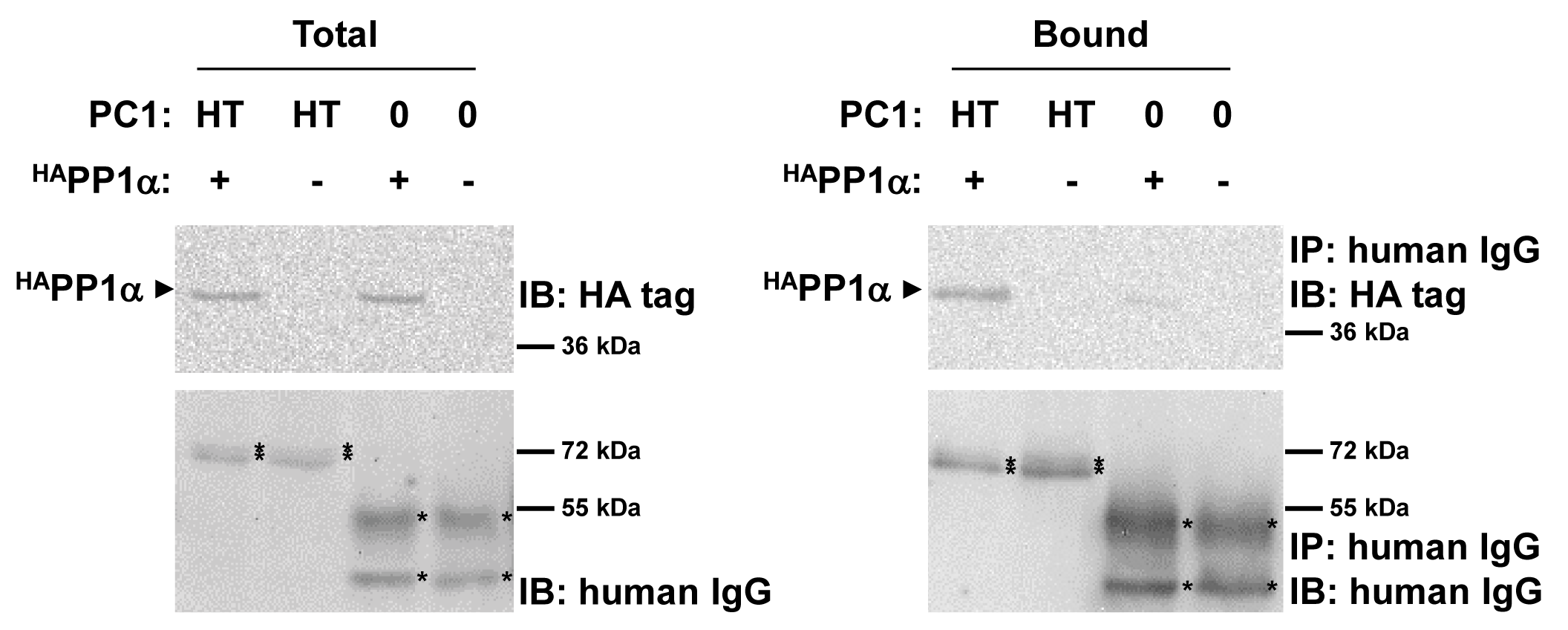

Supplement: Figure S2 — PP1α immunoprecipitates with PC1. 293T cells were transfected with plasmids encoding hemeagglutin (HA) epitope-tagged PP1α (HAPP1α) and the C-terminal, cytosolic 193 amino acids of PC1 fused to the membrane targeting cassette, sIg.7 [22]. Empty plasmid and a sIg construct lacking PC1 sequence (sIg-0) were used as controls for HAPP1α and sIg-HT193, respectively. Lysates from the transfected cells were immunoprecipitated (IP) with protein A/G+ agarose to pull down sIg- constructs. Bound and total fractions were resolved by SDS-PAGE and immunoblotted (IB) with anti-HA antibodies to detect HAPP1α, then stripped and re-probed with anti-human IgG Fc-alkaline phosphatase conjugated antibody to detect sIg-constructs. While some HAPP1α could be detected in the sIg-0 bound fractions, we consistently observed enrichment of HAPP1α when precipitated with sIg-HT193. (TIF) [file pone.0036798.s002.tif]

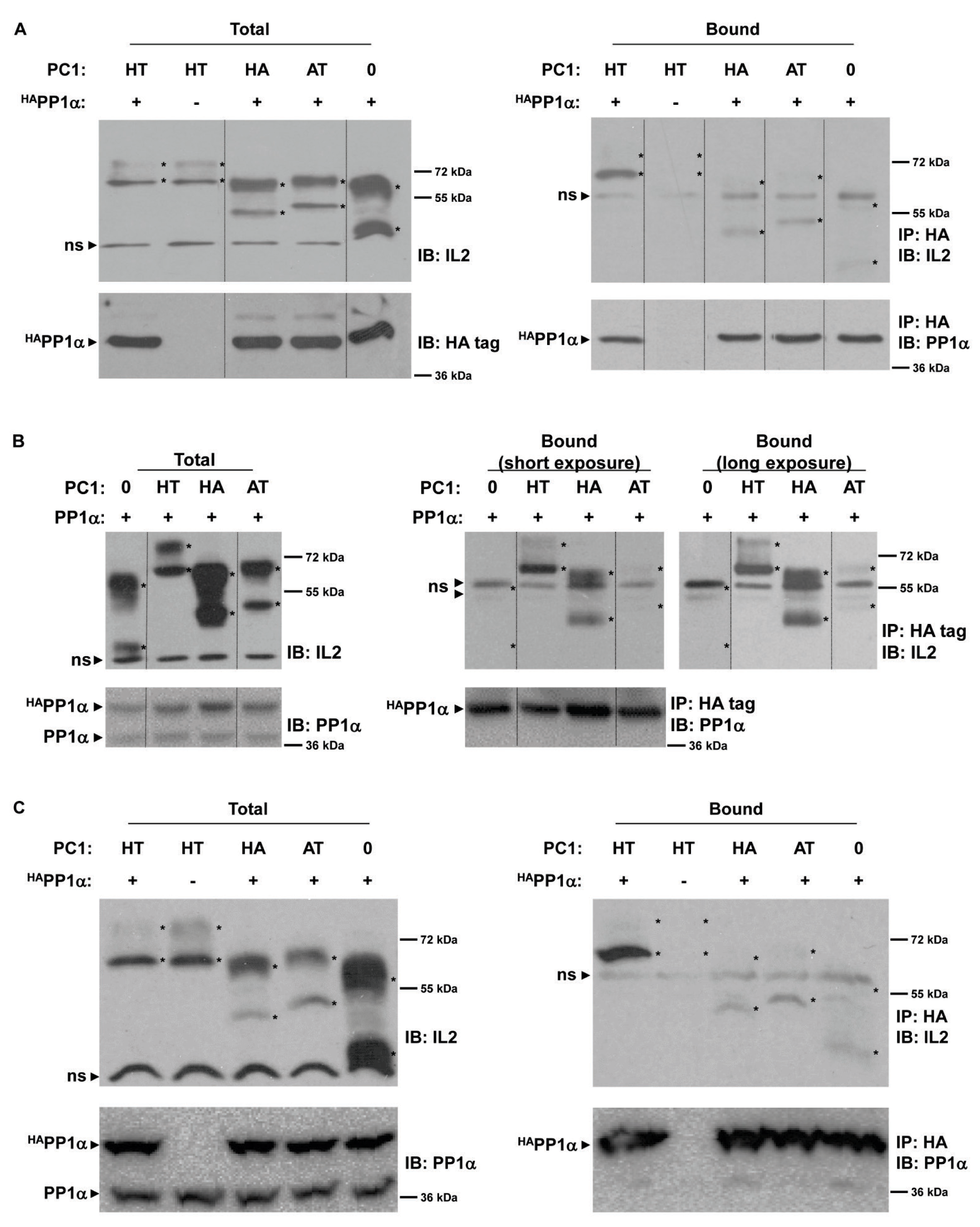

Supplement: Figure S3 — PC1 immunoprecipitates with PP1α. Panels A, B, and C are replicate co-immunoprecipitation experiments (see Figure 2). Note in A and B that there is less PP1α present in the IL2-HT193 bound fraction as compared to all other lanes, and yet there is significantly more IL2-HT193 co-immunoprecipitated than IL2-HA74, -AT120, or −0. Also note that more IL2-HT193 can be detected in the bound fraction of B despite dramatic over-expression of IL2-HA74 as demonstrated in the total fraction. In A and C, some IL2-0 can be detected in the bound fraction. However, the amount of IL2-0 in the bound fraction is never greater than that of any PC1 protein, and the amount of IL2-0 present in the total fraction is significantly greater than the amount of any PC1 protein. No IL2-0 was detected in the bound fraction of B. ns = non-specific band. (TIF) [file pone.0036798.s003.tif]

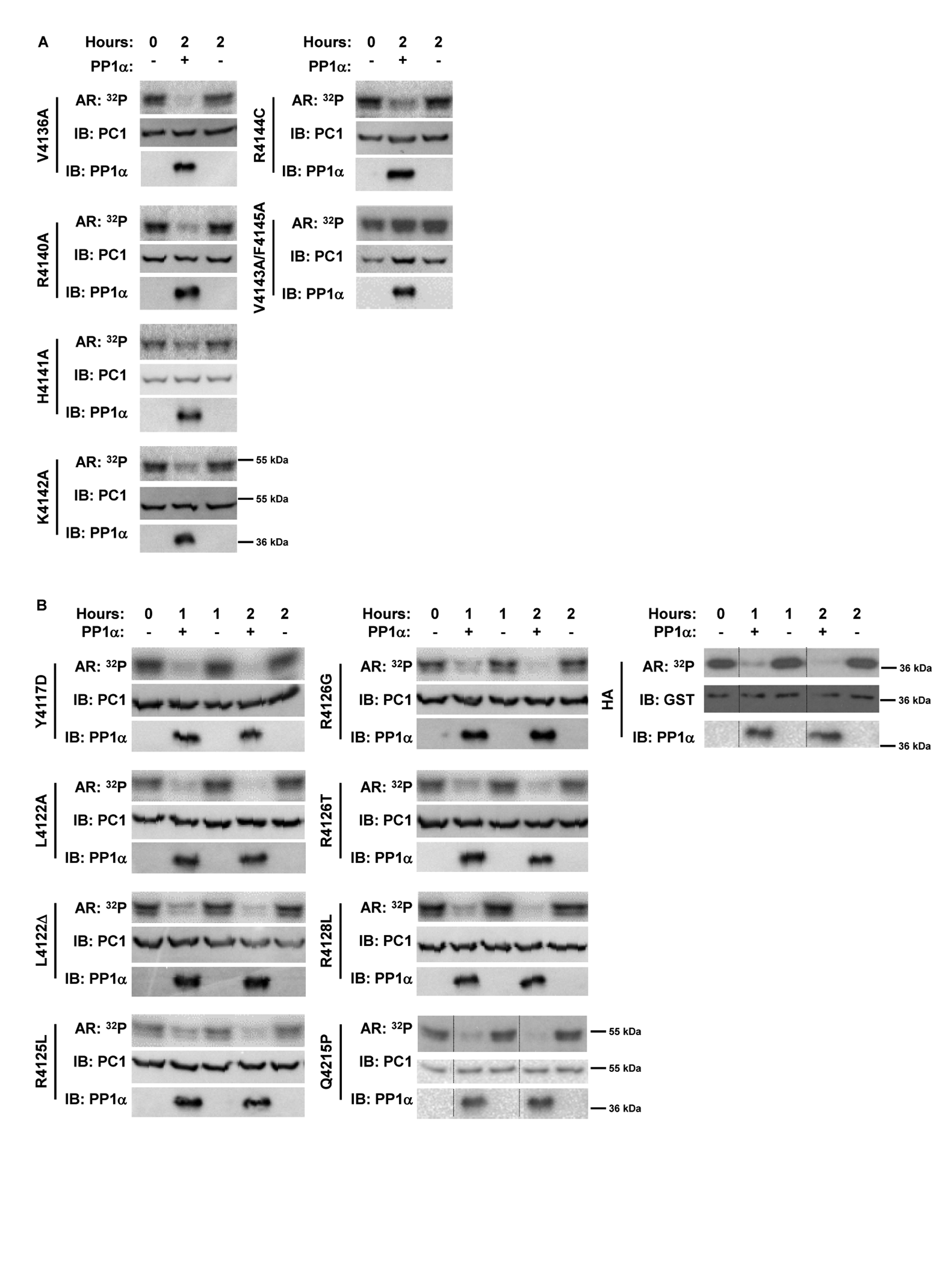

Supplement: Figure S4 — Representative autoradiographs and immunoblots of in vitro kinase/phosphatase assays. (A) Analysis of PC1 mutants V4136A, R4140A, H4141A, K4142A, R4144C, and the double mutant V4143A/F4145A was conducted as described in Figure 5. (B) Additional PC1 mutants as well as PC1 truncation protein HA74 were analyzed by autoradiography and immunoblotting as described in Figure 5. Mutations L4122Δ, R4125L, R4126G, R4126T, and R4128L are either naturally occurring PKD associated mutations and/or lie in or near the upstream FxxBxB site in PC1 (Figure 1A) [6], [7], [31], [49]. In addition, we had previously demonstrated that mutations L4122Δ, R4126G, and R4126T inhibit heterotrimeric G protein signaling (see Magenheimer, B. S., et al. (2002) J Am Soc Nephrol 13, 18A F-FC085). Q4215P is a PKD associated mutation that interrupts the coiled-coil domain of PC1 [50]. Y4117D was examined as it is a potential site of tyrosine phosphorylation [17]. GST-HA74 was examined because IL2-HA74 co-immunoprecipitates poorly with PP1α from lysates of co-transfected 293T cells (Figure 3). (TIF) [file pone.0036798.s004.tif]

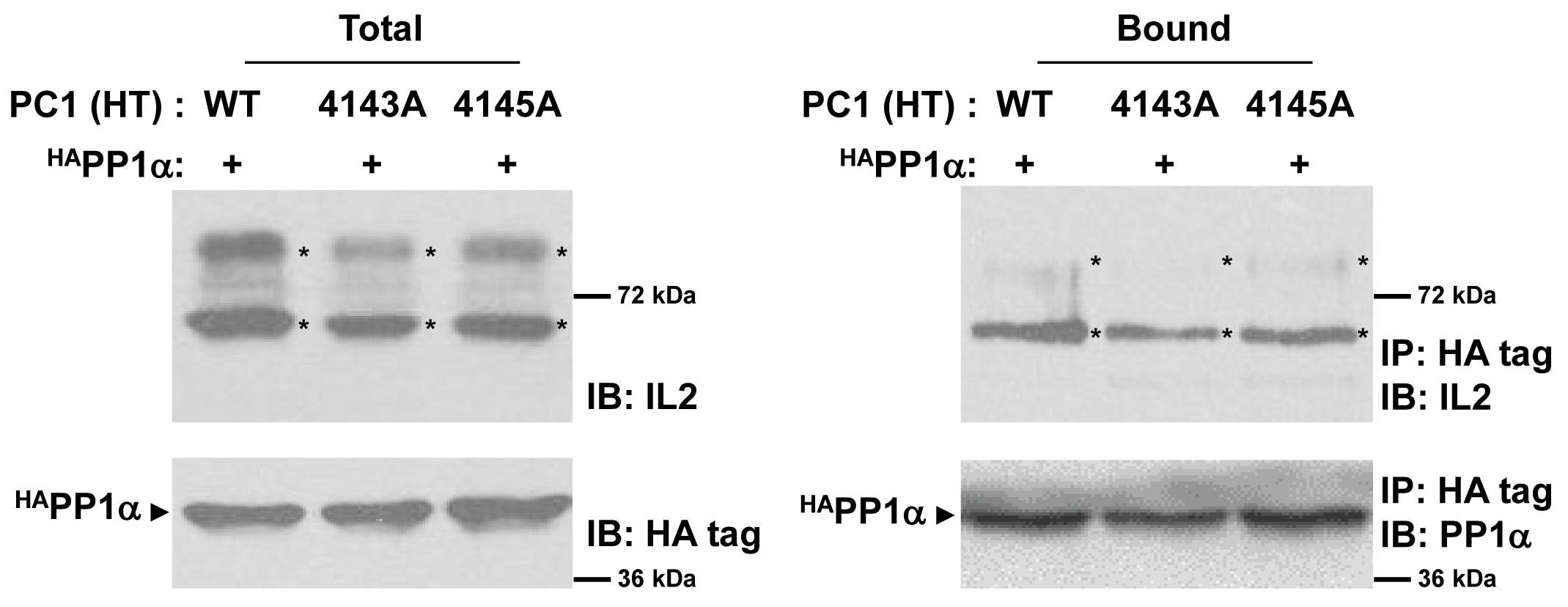

Supplement: Figure S5 — RVxF mutations fail to disrupt the PC1-PP1α interaction. To determine whether RVxF mutations disrupt binding between PC1 and PP1, 293T cells were transfected and immunoprecipitated as described in Figure 2 but with wild type and RVxF-mutated IL2-HT193 fusion proteins. RVxF mutations V4143A and F4145A failed to appreciably decrease binding with HAPP1α. (TIF) [file pone.0036798.s005.tif]
